# Mechanisms of propranolol action in infantile hemangioma

**DOI:** 10.4161/19381980.2014.979699

**Published:** 2015-01-26

**Authors:** Jina JY Kum, Zia A Khan

**Affiliations:** 1 Department of Pathology and Laboratory Medicine; Schulich School of Medicine & Dentistry; Western University; London, Ontario Canada; 2 Metabolism and Diabetes Research Program; Lawson Health Research Institute; London, Ontario Canada; 3 Division of Genetics and Development; Children's Hospital Research Institute; London, Ontario Canada

**Keywords:** adrenergic receptors, angiogenesis, apoptosis, endothelial cells, infantile hemangioma, propranolol, stem cells

## Abstract

Infantile hemangioma is a common tumor of infancy. Although most hemangiomas spontaneously regress, treatment is indicated based on complications, risk to organ development and function, and disfigurement. The serendipitous discovery of propranolol, a non-selective β-adrenergic receptor blocker, as an effective means to regress hemangiomas has made this a first-line therapy for hemangioma patients. Propranolol has shown remarkable response rates. There are, however, some adverse effects, which include changes in sleep, acrocyanosis, hypotension, and hypoglycemia. Over the last few years, researchers have focused on understanding the mechanisms by which propranolol causes hemangioma regression. This has entailed study of cultured vascular endothelial cells including endothelial cells isolated from hemangioma patients. In this article, we review recent studies offering potential mechanisms of how various cell types found in hemangioma may respond to propranolol.

## Abbreviations

Akt: protein kinase B
ADR: adrenergic receptor
bm-MPC: bone marrow mesenchymal progenitor cell
cAMP: cyclic adenosine monophosphate
CREB: cAMP response element binding protein
EC: endothelial cell; EPAC, exchange protein activated by adenylyl cyclase
Glut-1: glucose transporter-1
hemECs: hemangioma-derived endothelial cells
hemSCs: hemangioma-derived stem cells
hemPericytes: hemangioma-derived pericytes
IH: infantile hemangioma
IL: interleukin
MAPK: mitogen-activated protein kinase
MMP: matrix metalloproteinase
mTOR: mammalian target of rapamycin
PKA: protein kinase A
PPAR: peroxisome proliferator-activated receptor
VEGF: vascular endothelial growth factor

## Infantile Hemangioma

Infantile hemangioma (IH) is the most common vascular tumor of infancy.^1^ For reasons unknown, IH affects more females than males, and is also more prevalent in premature and Caucasian babies, ultimately affecting up to 10% of infants.^2,3^ IH is often noticed soon after birth, when a bright red lesion appears.^4^ Approximately 80% of these lesions are found in the head and neck regions, but they can be located elsewhere in the body.^5,6^ It has been well-established that IH follows 3 developmental phases.^7,8^ The first phase entails expansion of undifferentiated stem/progenitor cells.^9,10^ These stem/progenitor cells differentiate into atypical vascular endothelial cells (ECs) characteristic of IH. Uniquely, IH endothelium exhibits robust expression of glucose transporter-1 (Glut-1).^11,12^ This proliferating stage is completed by 8 months of age in most cases.^10^ In the following involuting phase, the differentiation process continues as hemangioma-initiating cells differentiate into ECs and pericytes.^13,14^ This involuting phase, like the proliferating phase, is a continuum of cellular and molecular changes with the end result being appearance of adipocytes and fibrofatty residuum.^7,8,14^ Most IHs resolve spontaneously and do not require treatment.^15^ However, therapeutic intervention is necessary in cases where the lesion grows in certain locations and to sizes that could result in life-threatening complications. An example of such a situation is the growth of IH in the airway to obstruct the respiratory system.^16^

## Current Treatment Options for IH

Although a number of attempts have been made, the treatment guidelines for IH are not fully clear due to the differential effects of various therapeutic options, the differences in the location, stage and size of the tumors, and the age of patients.^15^ Treatment is typically initiated during the early proliferative stage of the tumor at which point many treatment options are available such as surgery, laser, and corticosteroids.^17-20^ Corticosteroids represented a common treatment option for IH patients, however, there are severe side effects including severe growth retardation in children when used over an extended period of time at high doses.^21^ Propranolol, a synthetic β-adrenergic receptor (ADR) antagonist that is widely used to treat myocardial complications, was accidentally discovered to be a promising treatment for IH.^22^ This non-selective β-blocker proved to be more effective with fewer adverse events when compared to corticosteroids, such that it is now the first-line treatment option.^23^ Despite its effectiveness, a major challenge remains in understanding the therapeutic mechanism of propranolol in regressing IH.

Propranolol usage has shown remarkable efficacy.^23^ There are some adverse effects associated with propranolol use, which include sleep disturbances, acrocyanosis, hypotension, and hypoglycemia.^24,25^ In addition, there are also reports of IH regrowth following cessation of treatment in as many as 20% of the cases.^26,27^ Therefore, greater understanding of the potential mechanisms underlying the therapeutic effect is needed to develop better and safer treatment options. Many mechanisms have been proposed, though only tested in culture studies, to explain the therapeutic mechanism of propranolol in treating IH. Theories involving vasoconstriction,^28^ EC apoptosis via β-ADR signaling^29,30^ and the caspase pathway,^31,32^ and inhibition of angiogenesis via the modulation of vascular growth factors^30,33,34^ have been suggested. This review will emphasize the diverse mechanisms implicated with the therapeutic action of propranolol in the various cell types found in IH.

## β-adrenergic Receptor Signaling

β-adrenergic receptors (β-ADRs) are a family of G protein-coupled receptors that mediate physiological responses to adrenaline and noradrenaline. To date, 3 subtypes of β-ADRs have been identified: β1-3 ADRs. Although a putative β4 subtype has been suggested, the function and localization remains unknown. There is limited information available on β1-3 ADR distribution at the cellular as well as the tissue level. Highest levels of β1-ADR are believed to be found in the heart and brain.^35^ β2-ADR shows a wide spread distribution pattern.^36^ β3-ADR is believed to be predominantly expressed in adipose tissue consistent with its lipolytic function.^37^ In the blood vessels, studies have utilized β-ADR antagonist binding and shown sites in all cellular layers of vessels.^38,39^ Predominant ADR subtypes in vessels include β1 and β2 as confirmed by β-ADR subtype knockout studies.^38^ Using immunohistochemistry, β1 and β2 ADR protein has been localized to IH endothelium (co-localized to CD31-positive cells) as well as perivascular cells (co-localized to α-smooth muscle actin-positive cells).^40,41^ In addition, β3 ADR has also been reported in all phases of IH.^34^ Given that β-ADRs are present in normal vessels and IH vessels, the question arises as to the role of β-ADRs in vessel function and the effect of β-ADR blockade in IH resolution.

**Figure 1.**
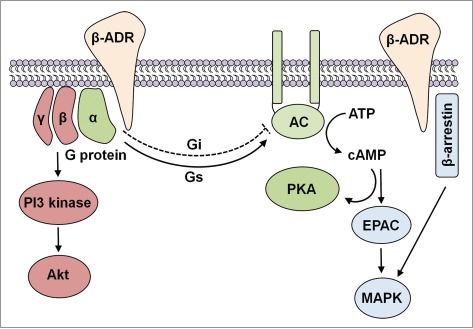
Schematic illustrating -adrenergic receptor signaling. Ligand binding to β-adrenergic receptors (β-ADRs) results in Gs-mediated activation of adenylyl cyclase (AC) and subsequent conversion of ATP into cAMP. Intracellular cAMP activates PKA to phosphorylate multiple target proteins. cAMP may also activate exchange protein activated by adenylyl cyclase (EPAC) leading mitogen-activated protein kinase signaling pathway and downstream effects on cellular processes. Another pathway activated by β-ADRs is the PI3 kinase and protein kinase B (Akt) pathway which may be initiated through dissociated Gβγ complex. In addition to G protein-mediated signaling, β-ADRs may also participate in G protein-independent signaling through β-arrestin and MAPK.

β1 and β3 generally couple with Gs (stimulatory) proteins, whereas β2 may couple with Gs or Gi (inhibitory) (**Fig. 1**). In the unstimulated state, the trimeric G protein is bound to GDP. Activation of ADRs promotes exchange of GDP for GTP. The G protein's α subunit with bound GTP then dissociates from the β and γ subunits to phosphorylate adenylate cycles (AC) and increase intracellular cyclic adenosine monophosphate (cAMP) levels. Gi may counteract this increase by inhibiting AC. Intracellular cAMP activates cAMP-dependent protein kinase A (PKA) which may have multiple cellular consequences.^42^ For example, PKA has been shown to be involved in elaboration of angiogenic factors through cAMP response element-binding protein (CREB).^43^ In addition to PKA-mediated signaling, activated AC may also activate mitogen activated protein kinase (MAPK) pathway through exchange protein activated by adenylyl cyclase (EPAC).^44,45^ Dissociated Gβγ may also lead to activation of phosphatidylinositol-4,5-bisphosphate 3-kinase (PI3 kinase) and Akt/protein kinase B. In addition, ADR signaling may entail a G protein independent signaling pathway. A well-characterized example is the β-arrestin-mediated activation of MAPK pathways.^46,47^ These pathways indicate that inhibiting β-ADR by propranolol in IH may, indeed, have beneficial effects by reducing cell survival as well as inhibiting angiogenesis. In support, Zhang et al have shown that propranolol does regulate MAPK pathway and activity of CREB in pancreatic cancer cells.^48^

## Cell-type Dependent Effect of Propranolol in Infantile Hemangioma

### Hemangioma-derived endothelial cells (HemECs)

Culture of hemangioma-derived ECs (HemECs) in the presence of propranolol causes apoptosis.^49^ This is evident upon exposure to 100 μmol/L propranolol in the culture media.^32,49,50^ Ji et al. also found significant increases in caspase-3 and -9 cleavage products, but not caspase-8 cleavage following propranolol exposure.^49^ These results are suggestive of an intrinsic apoptotic pathway mediated by propranolol. However, other studies have found an increase in protein and mRNA levels of caspase-8^32^, indicative of both intrinsic and extrinsic involvement of the apoptotic pathway. At the mRNA level, it was reported that propranolol induces expression of apoptotic genes, such as Bax, p53, caspase-8, and cytochrome c in hemECs that may be responsible for its apoptotic effect.^32,49^ It should be noted that these studies have essentially examined the effect of propranolol without the addition of β-ADR stimulation. This may suggest a constitutively active β-ADR pathway. Stress hormones or growth factors in serum may also play a role. Interestingly, propranolol's effect is not specific to hemECs, as it has been shown to cause apoptosis in a similar manner with other EC types as well.^29,50,51^

Many studies have gone in-depth with analyzing the expression levels of the different β-ADR subtypes. It has been shown that hemECs and other EC types express both β1- and β2-ADRs at very similar levels, but not β3.^52,53^ We have shown that normal ECs express all 3 β-ADRs, with β1-ADR expression being significantly higher when compared to the other subtypes.^50^ Despite the various β-ADRs expressed, it is believed that the main mechanism of action of propranolol in hemECs may involve β1 and/or 2-ADR pathway. A recent report by Ji et al. have shown that ICI-118551 (a selective β2-ADR antagonist) was more effective than metaprolol (selective β1-antagonist) in inhibiting hemECs proliferation.^54^

In addition to caspase-mediated apoptosis, propranolol may block phosphorylation of vascular endothelial growth factor receptor 2 (VEGFR-2).^52^ It was found that when hemECs were challenged with higher concentrations of propranolol (50 and 100 μmol/L), the expression of VEGF at the protein level was reduced in a dose-dependent manner.^49,55^ This reduction in the level of activated VEGFR-2 receptors and VEGF protein upon propranolol exposure was a critical element that affected the survivability of these hemECs.^52,56^ In addition, decrease in key cyclin levels and an increase in cell cycle inhibitor levels were observed.^52^ This suggested that cell cycle regulation is also another mechanism involved in mediating propranolol's therapeutic effect. HemECs show a greater proportion of cells in the G1 phase than the S/G2 phase when treated with propranolol.^29,52^ This was further confirmed with decreased expression of cyclin proteins such as cyclins A1, A2, B2, D1, D2, D3,^29,50,52^ while cell cycle inhibitor proteins p15, p21, p27,^52^ were up-regulated.

### Hemangioma-derived stem cells (HemSCs)

We have shown that IHs are derived from multipotential stem cells termed hemangioma stem cells (hemSCs).^9^ Clonally expanded hemSCs produce Glut-1 positive microvessels in immunodeficient mice. Boscolo and colleagues have shown that binding of VEGF-A and VEGF-B to VEGFR-1 expressed on the surface of hemSCs is required for the induction of hemSC to EC differentiation, and for blood vessel formation.^57^ Interestingly, immunostaining of IH specimens shows co-labeling of EC markers and stem cell markers indicative of an immature EC phenotype in IH.^14,50^ Since IHs regrow in a significant proportion of patients that discontinue propranolol treatment,^26^ it is possible that hemSCs, unlike hemECs and normal vascular ECs, are not susceptible to propranolol-induced apoptosis. This may explain why recurrence of these tumors often occurs following cessation of treatment. We have recently shown that propranolol causes significantly reduced cell number.^50^ To determine whether the decrease in hemSCs was due to changes in the cell viability as expected from studies utilizing ECs, caspase-3 was measured. Unlike ECs, the levels of activated caspase-3 were significantly reduced in hemSCs.^50^ This indicates that hemSCs do not undergo apoptosis upon propranolol exposure. However, propranolol exposure significantly decreased cyclin-D1 levels in hemSCs, similar to its effect in ECs,^50^ suggesting that the decrease in hemSCs cell number upon propranolol exposure is not due to apoptosis, but rather the inhibition of cell cycle progression. Furthermore, Zhang and colleagues have shown that propranolol reduces VEGF expression in hemSCs, that later suppresses angiogenesis.^58^ At the protein level, the quantity of VEGF produced by hemSCs decreased in a dose-dependent manner, showing a significant reduction even at a very low concentration (0.02 μmol/L) of propranolol.^58^ This decrease in VEGF levels from hemSCs may also contribute to their quiescent state, rather than apoptosis, upon propranolol treatment.

The difference in propranolol response between hemECs and hemSCs may be related to β-ADR subtype expression. Studies have shown that β1-ADR and β2-ADR have opposing effects on regulating apoptosis.^59-61^ HemSCs express β2 and β3-ADRs, whereas ECs predominantly express β1-ADR.^50^ This suggests that propranolol may mediate its apoptotic effect in ECs through β1-ADR, and cell cycle arrest in hemSCs through β2-ADR. A novel mechanism we have observed in hemSCs is a significant induction of anti-apoptotic genes following exposure to propranolol.^50^ Although these inductions were significantly higher in hemSCs, anti-apoptotic genes were also evident, but to a lesser degree, in normal bone marrow-derived mesenchymal progenitor cells (bm-MPCs).^50^ This indicated that upon propranolol exposure, these stem/progenitor cells trigger a mechanism that induces anti-apoptotic genes to provide these cell types with an apoptotic escape route, unlike ECs.

IH ends its continuous developmental phase when adipocytes replace majority of the tumor lesion. Yu and colleagues first reported presence of cells with adipogenic differentiation potential in proliferating phase IH.^62^ More recently, we have shown that clonally derived hemSCs produce human adipocytes when implanted in immunodeficient mice.^9^ Culturing hemSCs in the presence of propranolol enhances adipogenesis and this may offer another possible mechanism of the beneficial effects of propranolol in IH resolution. Continuous culture of hemSCs in adipogenic differentiation media supplemented with propranolol causes cell death.^31^ These results suggested that propranolol treatment accelerated the dysregulated differentiation process in hemSCs that ultimately resulted in increased apoptosis of adipocytes derived from hemSCs.^31^ It is possible that differentiation of hemSCs causes a shift in β-ADR expression profile and an increase in β1-ADR which may induce apoptosis. We have found that upon adipogenic differentiation in hemSCs, all 3 β-ADR subtypes are significantly increased. Therefore, changing β-ADR profile may increase cell's susceptibility to apoptosis, in a manner similar to that of propranolol-treated ECs.

### Hemangioma-derived pericytes (HemPericytes)

Pericytes are cells that control EC proliferation and survival by stabilizing the vasculature wall and releasing pro-survival signals.^63^ In IH, pericytes are also believed to be derived from hemSCs.^64^ When hemSCs are in direct cell contact with ECs, hemSCs can also differentiate into pericytes through the JAGGED1 signaling.^64^ It is known that addition of β-ADR agonists and cAMP analogs can induce relaxation of pericytes.^65^ When hemPericytes were exposed to propranolol, epinephrine-induced relaxation in hemPericytes was prevented.^66^ Furthermore, the proliferative capacity of hemPericytes was also reduced. These pericytes also expressed β2-ADRs on their cell surface.^66^ With knockdown of β2-ADR, hemPericytes lost epinephrine-induced relaxation and propranolol had no effect.^66^ This suggested that β2-ADR is involved with relaxation and contractility of hemPericytes in response to propranolol.^66^ In addition, when hemPericytes co-implanted with hemECs were exposed to propranolol, propranolol decreased the vascular volume indicative of increased vasoconstriction.^66^ This may be suggestive of a possible mechanism by which propranolol causes increased constriction of the vasculature in IH to reduce the blood flow to the tumor, limiting its growth.

## Effect of Propranolol in Other Human Neoplasms

The progression of various cancers has been associated with alteration of β-ADR signaling pathways. Hence, β-blockers have been proposed as therapeutic agents for various cancers. Pediatric melanoma is a rare disease but its incidence has increased in the young population. Melanoma is accountable for up to 3% of all pediatric malignancies.^67^ Similarly to IH, melanoma cases are more commonly diagnosed in Caucasian and female patients,^68^ and almost 20% of malignant melanoma occurs in the head and neck region.^69^ Melanocyte stem cells (MSCs) generate melanocytes that produce melanin-pigment throughout adult life. When MSCs undergo mutation and transform, it can result in melanoma.^70,71^ The pathogenesis of melanoma is still subject to debate, but many have suggested mutations involving the cell cycle and apoptosis pathways, such as tumor protein p53 pathways, and stressors that increase catecholamines are involved in tumor progression.^72,73^

The increase in norepinephrine and epinephrine primarily modulates the β-ADR pathways, through PKA and MAPK signaling mechanisms, ultimately affecting the growth and progression of melanoma.^74^ Additionally, increases in the expression of VEGF, interleukin (IL) -6 and IL-8 after catecholamine stimulation correlates with the aggressiveness of the tumor.^74,75^ Similar to IH, melanoma cells express β1 and β2-receptors with β1-ADR expression being weaker relative to β2-ADR.^72,74^ Recently, β3-ADRs have been proposed to be involved with melanoma growth and vascularization.^76^ The use of β-blockers in malignant melanoma decreased the risk of progression.^77^ Although the exact mechanism underlying the effectiveness of these medications in reducing tumor progression is unknown, it has been suggested that β-blockers reduce angiogenic factors and metastatic progression.^77^ It is thought that β-blockers inhibit angiogenesis by reducing VEGF activity via MAPK signaling. In addition, β-blockers also modulate matrix metalloproteinases (MMPs) that can alter the tumor microenvironment involved with angiogenesis to further inhibit the formation of new blood vessels.^78^ Recently, specific inhibition of β3-ADRs in melanoma cells was found to impair cell growth and induce apoptosis.^76^

β-ADR has also been implicated in breast cancer. Breast cancer cells express both β1- and β2- ADRs,^79,80^ and the polymorphisms of β-ADR subtypes may be associated with breast cancer susceptibility.^81^ Breast cancer patients who received propranolol for hypertension displayed reduced metastasis and cancer recurrence.^82^ This may be due β-ADR signaling involvement that alters gene expression within the primary tumor.^83^ Further investigation of β-ADR signaling provided evidence that the β-ADR pathway controls the stimulation of the arachidonic acid cascade.^84^ In breast cancer development, arachidonic acid is a critical molecule that has been shown to activate mTOR and increase the activity of VEGF.^85^ mTOR and VEGF seem to be a common pathway in breast cancer and in hemECs, involving enhanced angiogenesis.

## Direct and Indirect Effects of Propranolol

Although propranolol treatment and β-ADR antagonism seems promising for IH as well as a number of other human cancers, there is quite a bit of knowledge gap. This essentially involves understanding whether propranolol mediates the effects through blocking β-ADR or another indirect mechanism. There are possibilities that need to be explored: 1) involvement of receptor dimerization and α-ADR signaling, and 2) involvement of serotonin (5HT) signaling. Although data is limited, propranolol does stereoselectively bind and inhibit α-ADR in the heart.^86^ Immunoprecipitation studies also show that β1-ADR and α2-ADR heterodimerize when co-expressed.^87^ This interaction changes the pharmacological properties of β1-ADR as shown by ligand binding assays. β1- and β2- ADR have also been shown to heterodimerize.^88,89^ These findings suggest that the profile of ADR may have functional cellular consequence and represents an area of significant future research interest. In addition to ADRs, propranolol may also mediate its effects through serotonin (5HT) receptors.^90,91^ The interaction between propranolol and 5HT occurs with high affinity as well as low affinity 5HT receptors.^90^ There is also experimental evidence that propranolol acts as a 5HT1A antagonist and a 5HT1B agonist in the rat cortex.^92^ Treatment with propranolol also inhibited basal cAMP and steroidogenesis in rat leydig cells, with effects evident at 0.1 μmol/L.^91^

## Concluding Remarks

Propranolol has shown promising effects in IH resolution and many studies have sought to understand the mechanism of propranolol as an effective treatment. Recent clinical studies have also suggested that the use of β-blockers is effective in treating several tumors and cancers. β-ADR subtypes are associated with cancer growth and progression by increasing angiogenic, migratory, and invasive factors in tumor cells. In culture studies, propranolol causes apoptosis in ECs, as well as reduces VEGF to decrease angiogenesis. However, different responses are seen in perivascular cells and hemangioma derived stem cells. β-ADR-associated proteins may exhibit distinct tissue localization and underlie the differential activity of propranolol. Identification of cytoplasmic regulatory proteins in IH-derived cells that interact with β-ADRs may represent an attractive future research area for the development of cell-type specific therapies.
